# Cell Proliferation in Cutaneous Malignant Melanoma: Relationship with Neoplastic Progression

**DOI:** 10.5402/2012/828146

**Published:** 2012-01-11

**Authors:** G. E. Piérard

**Affiliations:** Department of Dermatopathology, University Hospital of Liège, 4000 Liège, Belgium

## Abstract

The establishment of the diagnosis of cutaneous malignant melanoma (CMM) always calls for histopathological confirmation. Further to the recognition of the CMM aspects, immunohistochemistry is helpful, in particular, in determining the size of the replicative compartment and the activity in each of the cell cycle phases (G_1_, S, G_2_, M). The involvement of cancer stem cells and transient amplifier cells in CMM genesis is beyond doubt. The proliferation activity is indicative of the neoplastic progression and is often related to the clinical growth rate of the neoplasm. It allows to distinguish high-risk CMM commonly showing a high growth rate, from those CMMs of lower malignancy associated with a more limited growth rate. The recruitment and progression of CMM cells in the cell cycle of proliferation depend on mitogen-activated protein kinase (MAPK) pathway and result from a loss of control normally involving a series of key regulatory cyclins. In addition, the apoptotic pathways potentially counteracting any excess in proliferative activity are out of the dependency of specific regulatory molecular mechanisms. Key molecular components involved in the deregulation of the growth fraction, the cell cycle phases of proliferation, and apoptosis are presently described in CMM.

## 1. Introduction

For years, the incidence of human cutaneous malignant melanoma (CMM) is steadily on the rise [1]. CMM represents the leading cause of skin cancer death in the Caucasian populations from Westernized societies [2, 3]. Survival is dramatically decreased when distant metastases develop. Mortality rates vary by country, gender, and ethnic origin.

For decades, physicians searched for predictive indicators of the CMM metastatic risk. The regular classification and staging of sporadic CMM rely on the combination of gross clinical and microscopic aspects [4, 5]. However, in some instances, the distinction of CMM from other atypical melanocytic neoplasms and the CMM staging prove to be difficult or uncertain [6]. The refinement of diagnostic assessments benefits from complementary immunohistochemical investigations [7–12]. Recent progress in the field of molecular biology brings further information resolving a series of translational quandaries [13–16]. In particular, it appears that transient amplifier neoplastic melanocytes, as well as melanocytic stem cells, participate in the CMM initiation and progression [15, 17–24].

Basically, most CMMs begin with the so-called radial growth phase corresponding to a slow-growing in situ and microinvasive phase. This condition is characterized by a high cure rate. Despite a trend toward earlier clinical recognition of CMM, by the time of diagnosis, most of the CMMs have already progressed to the next vertical growth phase typical for tumorigenic CMM and characterized by a faster growth rate [24]. In these neoplasms, cure becomes uncertain, and the prognosis depends on certain attributes of both the neoplasm and the host (stroma, immunity, etc.).

In general, the CMM clinical progression appears in part correlated with the enlargement of its germinative compartment [23, 24]. The proportion of CMM cells engaged in the cell cycle of proliferation is increasing as well. However, some cases of smouldering CMM, CMM dormancy, and CMM regression exhibit peculiar aspects of tumor progression and cell proliferation [25–27].

## 2. Smouldering CMM

A variety of interrelations between CMM cells and the host are under the control of a vast array of factors. These interactions are not static, but result from a fluctuating imbalance between the neoplasm and the host, both being engaged in a process of natural selection. The variable combination over time of different cell processes involving CMM and its microenvironment possibly leads to a condition coined smouldering CMM [27].

 The smouldering CMM concept was raised following a seminal study on CMM performed over more than 20 years [25]. The observational study scrutinized how the CMM metastases possibly varied in their evolution in each individual. The smouldering CMM phenomenon was defined as metastases unexpectedly appearing and disappearing on the same body region over months and years. In their wax and wane progression the metastases usually reached at the most the size of a pea or a bean [25]. Such condition was likely associated with contrasted active cell proliferation and apoptosis/necrosis.

 The smouldering CMM phenomenon contrasts with the more common surge appearance of metastatic crops. This latter condition was particularly reported following excision of some primary CMM at a time when the metastatic disease remained silent and undisclosed. In short, smouldering CMM highlights the fact that CMM progression is not a fixed process and an ineluctable feature. Indeed, the net growth directionality of CMM metastasis proceeds through a combination between growth and regression. This evolution is influenced by (a) the possible prevalence of apoptosis over proliferation, (b) the versatile antitumoral immunity, and (c) the failure of stromal/vascular receptivity. Of note, inflammation in a variety of neoplasms boosts (a) the proliferation and survival of malignant cells, (b) angiogenesis, (c) metastasis release, and (d) subversion of adaptive immunity. In addition, inflammation potentially alters the response of the neoplasm to hormones and various chemotherapeutic agents [28]. Such a multifaceted process possibly leads to a programmed pathway of apoptosis and necrosis.

## 3. CMM Dormancy

Globally near 40% of CMM patients develop clinical metastases 5 years or so after initial treatment. However, the disease-free interval before appearance of metastases possibly elapses 10 to 25 years or over. Any unusually long latency period between the primary CMM treatment and metastatic occurrence is commonly thought to result from clinical CMM dormancy [27]. It is associated with a protracted progression-free survival. The relationship between the CMM clinical dormancy and the CMM cell dormancy is complex and probably multifactorial. The neoplastic quiescence process is typically unstable and possibly leads to CMM relapse [29]. Any delay in CMM metastases suggests the implication of some host defence mechanism, and/or a peculiar nature of non- or slow-proliferating CMM cells possibly involving CMM stem cells [27].

 Neoplastic dormancy and autophagy are partly correlated. Autophagy is a homeostatic and catabolic process involving cytoplasmic organelles and proteins. The process enables particle sequestration and their lysosomal degradation. Overall, autophagy is a mechanism of stress tolerance maintaining cell viability and possibly leading to any aspect of tumoral dormancy, progression, and therapeutic resistance [30]. Such a process is important for the maintenance of genomic stability and cell survival.

A dramatic example of CMM dormancy deals with metastases developed from transplant organs in drug-immunocompromised recipients [31, 32]. Silent micrometastases present in the organ donor subject develop in an uncontrolled brisky way when the invaded organ is transplanted in the immunocompromised patient.

## 4. Growth Fraction and Cell Kinetics in CMM

Most cancers including CMM are composed of a number of different cell subpopulations expressing distinct cell kinetics, as well as atypical ploidies, different drug and radiation sensitivities, and variable metastatic potential. Whatever the source of cell heterogeneity, such cell diversity is present at different sites within each single neoplasm, at different metastatic locations in a given individual, and among patients exhibiting seemingly similar CMM types and stages.

The cell cycle of proliferation encompasses a sequential series of biochemical events leading to DNA replication and cell division [24]. The time elapsed between two successive cell divisions in a sustained dividing process corresponds to the cell cycle time (*T*_*c*_). After mitosis completion, the cell enters the G_1_-phase. At a specific restriction point in early G_1_-phase, the cell sets in motion a specific biochemical step allowing progression through an ordered sequence of other biochemical steps leading to DNA replication during the S-phase. Alternatively, the cell enters into the nondividing quiescent G_0_-stage, thereby reducing the growth fraction (GF) of replicating cells. Once DNA duplication is completed, the cell enters the G_2_-phase that ends the process required for cell division. At mitosis the cell divides into two daughter cells which possibly reenter the G_1_-phase for reinitiating another cell cycle.

GF of any tissue or any neoplasm corresponds to the proportion of the cell population actually engaged in the cell cycle of proliferation. Clearly, GF is reduced by any segment of the cell population in the resting G_0_-stage. In addition, cell loss from the dividing and nondividing compartments occurs through cell apoptosis and necrosis as well as through metastatic cell escape. Therefore, the rate of cancer growth depends on three factors, namely, the average *T*_*c*_, the GF, and the cell loss.

The time to proceed through the S-, G_2_- and M-phases of the cell cycle remains fairly constant in any given tissue. By contrast, the extent in the G_1_-phase is more variable, in part due to the different restriction points controlling the progress through the cell cycle or the derivation to the G_0_-phase. The variability in the length of G_1_-phase makes *T*_*c*_ frequently differ from cell to cell. Neoplastic cells generally exhibit a longer *T*_*c*_ than the corresponding normal cells. This characteristic is in part explained by the presence of CMM stem cells exhibiting a lengthy *T*_*c*_ [23]. The mechanisms ruling the proliferative activity (*P*) in a given tissue are linked to both GF and the speed rate of the cell cycle with *P* = *GF*∗*T*_*c*_^−1^. Thus, more of the GF and *T*_*c*_ singly defines the actual *P* of a neoplasm. As a rule, *T*_*c*_ is not accessible clinically and thus, *P* of CMM remains not measurable.

## 5. Immunohistochemistry and CMM Growth Fraction

Both tumoral cell renewal and GF are important aspects determining in part the CMM malignancy potential. Regular counting of mitotic figures is a time-honored method used for assessing cell proliferation, but its sensitivity and reliability remain quite low because their numbers are usually low. To fine-tune such evaluation, any immunohistochemical method exploring one single specific facet of cell renewal requires the restricted expression or a prominent cell-cycle-induced increase of a given target antigen in cycling cells. A number of antibodies to cell-cycle-related proteins fulfill such requirements. The target antigens belong to two distinct categories. One group comprises specific molecular compounds such as those involved in DNA synthesis during the S-phase. The second group encompasses cell-cycle-associated proteins, the most typical being represented by some histones and the MIB/Ki-67 nuclear antigen.

The proliferation marker MIB1/Ki-67 is produced and expressed during all active steps of the cell cycle of proliferation (G_1_-, S-, G_2_-, and M-phases), but its production is arrested in the G_0_-stage. The MIB1/Ki-67 monoclonal antibody provides a convenient means for evaluating the GF of CMM. The GF assessed by the Ki-67 index represents the ratio of cycling cells to the overall cycling and noncycling cells. Of note, cells that have run a part of the cell cycle before being rerouted at a restriction point to the G_0_-phase will retain the MIB/Ki-67 immunolabeling despite the fact they are no more part of the GF [23, 24]. In several malignancies, in particular CMM, the Ki-67 index is indicative of the neoplastic progression, and it appears as an important prognostic indicator [5, 7, 10–12, 23, 24, 33–39].

Globally, the findings about the GF of CMM support the clinical concept distinguishing CMM of high and low growth rates, each bearing a different prognosis [40–42]. Cancer registries suggested the existence of three unrelated CMM types recognized as (a) thick CMM, with stable incidence over the past decades, (b) thin CMM mainly located on the trunk, and showing a dramatic increase in incidence over the past years, and (c) CMM mainly located on the head and neck region showing a moderate trend in incidence increase over time [43, 44]. There is ample evidence that the GF extent in CMM is indicative of the neoplastic progression [13, 33–37, 45–49]. Ki-67 immunolabeling is positive in less than 5% of nevocytes in most melanocytic nevi, but it commonly raises up to 15% or so in benign atypical melanocytomas and it reaches 15–30% or more in CMM [5, 7, 10–12, 33–37, 47].

Any MIB1/Ki-67 index in CMM does not predict the number of cells completing mitosis. Indeed, all cycling cells including those arrested at a restriction point in the G_1_-phase are labeled. Hence, any fast-growing CMM is likely associated with a high MIB/Ki-67 index but a high MIB/Ki-67 index does not always imply a fast growing neoplasm.

 A stochastic relationship apparently prevails between CMM GF and tumor vascularity [38, 46]. Nevertheless, clinically growth-stunted CMM appeared to be generally associated with a restricted blood vascularization [47]. Thus, it was inferred that the extent in blood microvasculature and the GF size of CMM were mostly correlated when angiogenesis was restricted [47]. Of note, the CMM GF appears to be influenced by the intra- and peri-CMM infiltration by Factor XIIIa-positive dendrocytes [34, 50, 51]. There is circumstantial evidence linking the density of Factor XIIIa-positive dendrocytes and a low proliferative rate in CMM cells [34]. The biologic and molecular mechanisms supporting these findings remain unsettled.

In short, two main clinical applications of the melanocyte kinetics emerge, namely, (a) deciphering the distinction between benign melanocytic neoplasms and CMM and (b) establishing or refining a clinical prognosis for CMM progression [8, 23, 24, 34–37].

## 6. CMM Phase Indexes

Two phase indexes are of particular interest in CMM. They correspond to the S- and M-phases. Any phase index is defined as the proportion of cells engaged in that given phase of the cell cycle. Some of these cells are specifically recognized by morphology (mitosis), and others are possibly labeled (S-phase). There is no fixed correlation between the GF and any specific phase index. Indeed, the proportion of cells in a given cell cycle phase depends on both GF and the relative duration of that cycle phase (phase duration/*T*_*c*_). Different CMM cell populations, particularly their metastases, show different phase indexes due to (a) differences in their GF while the ratios between their respective phase durations to *T*_*c*_ are similar, (b) differences in the ratio of phase duration to *T*_*c*_ while their respective GF remains similar, (c) differences in the total phase duration while their respective replicative activity is similar, or (d) differences in their replicative activities while their phase durations are similar [23, 24]. Among distinct CMM exhibiting different phase indexes, the neoplasm exhibiting the higher value in a given phase index is not necessarily the most proliferative one.

A landmark study was performed 25 years ago using incorporations of tritiated thymidine (^3^H-TdR) [52]. CMM thickness appeared correlated with the proportion of CMM cells in the S-phase of proliferation. The higher ^3^H-TdR indexes were found in metastases.

The M-phase has long attracted attention in CMM. On a descriptive ground, finding mitotic figures in the deep portion of a melanocytic neoplasm was considered to be suggestive for CMM. Quantitative assessments were used to derive a prognostic factor (PF) following PF = M* tumor thickness where *M* represented the number of mitosis per mm^2^ [53]. More recently, a revival of mitosis interest emerged in CMM [54–56] and in melanocytic nevi as well [57]. Currently, it is possible to highlight mitoses using immunohistochemistry directed to specific *M*-associated histones. Indeed, DNA is wrapped around histone octamers to form the basic unit of chromatin structure. The octamer is composed of histones H2A, H2B, H3, and H4, and it associates with approximately 200 base pairs of DNA to form the nucleosome. Histone H3, the core protein of the nucleosome, becomes phosphorylated. The two major sites of phosphorylation are the M-specific site Ser 10 and Ser 28, both of which are extensively phosphorylated in DNA-bound forms of histone H3 and in nucleosomal histone H3. The p-histone H3 (Ser 10)-R detects Ser 10 phosphorylated histone H3 [58, 59].

## 7. CMM Stem Cells

In the past decades, CMM evolution was commonly described as the result of a “dedifferentiation” process of transformed mature melanocytes, enabling a stepwise metamorphosis starting from the radial growth phase, evolving toward the vertical growth phase, and ultimately leading to the disseminated disease. More recently, the heterogeneity and plasticity of the CMM cell populations led to the cancer stem cell (CSC) concept [60–67]. The regular stem cells of the melanocytic lineage are present in the human hair follicle, the epidermis, and the dermis [18, 68, 69]. The mutated melanocyte stem cells or immature melanocytic progenitor cells present in the skin were considered as possible precursors to CMM [17, 18, 20, 64].

CSCs, also referred to as tumor-initiating cells, were initially identified in several hematological malignancies and several solid cancers [60, 65]. Similar to physiologic postnatal stem cells, CSCs are capable of self-renewal and differentiation, and they are potentially involved in a perpetual self-renewal, a function sustaining unlimited tumor growth [61]. Although conventional anticancer treatments eradicate most of the target malignant cells, such drugs are typically inactive against chemoresistant CSC. This failure is ultimately responsible for neoplastic recurrence and progression. CMM exhibits cell heterogeneity, various molecular signatures, variable plasticity, and prominent tumorigenicity of some CMM cell subsets. These features are probably related to the involvement of CSC in the initiation and progression of the neoplasm [17–23, 61–67].

Embryonic and postnatal physiologic stem cells are capable of self-renewal at a slow cycling rate ensuring perpetual but discrete proliferation [61]. In addition, the multipotent stem cells possibly differentiate into cell types of multiple lineages, whereas unipotent stem cell give rise to a single lineage [17–19, 60]. The stem-cell-derived cancer model purports that most cancers are composed of relatively few CSCs steadily undergoing self-renewal, thereby maintaining a lifelong CSC pool. In addition, some other CSCs differentiate into fast-growing transient amplifier cells or progenitor cells, that in turn differentiate into mature cancer cells with limited proliferative potential [23, 70]. The latter cells form the bulk of the neoplastic GF in the primary CMM and its metastases.

Each CMM exhibits genetic and phenotypic heterogeneity, undifferentiated molecular signatures, and various developments of tumorigenicity and metastatic potential [11, 17, 71]. Primary CMM initiating cells appear restricted to CD133-positive (prominin-1) CSC, whereas CD133-negative CMM cells apparently lack tumorigenicity [70]. The embryonic-like differentiation plasticity supports the concept of involvement of CMM stem cells in the neoplastic evolution, from tumor initiation to progression and metastasis [71]. In addition, CMM contain some CSC-associated proteins as well as progenitor cell-specific molecules including cancer testis antigens [72] and bone morphogenetic proteins [73]. Genetic aberrations in the signal-transduction machinery of CSC survival and differentiation contribute to both CMM progression and metastatic spreading. The similarity of self-renewal mechanisms between physiologic stem cells and CSC [74] is a further argument suggesting the involvement of melanocytic stem cells in the CMM pathogenesis.

Chemotherapy resistance of advanced CMM, particularly in its metastatic stage, possibly results from several mechanisms linked to CMM stem cell biology, including the impairment of CMM apoptotic pathways, a relative proliferation quiescence, and a restricted intracellular drug accumulation. In particular, CMM cell dormancy [75] and CMM recurrence following initial remission is possibly related to the inability of current drug regimens to eradicate the putative compartment of CMM stem cells [64, 76–78].

## 8. Cyclins in CMM

Three optional physiologic pathways conceptually govern the cell life. Accordingly, cells continuously proliferate, or survive without further divisions, or die following apoptosis. The decision for a cell to proceed through the proliferative cycle is taken at two cardinal restriction checkpoints corresponding to the commitment to DNA replication and the commitment to mitosis at completion of the G_2_-phase. Throughout the G_1_-phase, a number of growth factors influence the cell fate through binding to specific surface receptors. This process results in cell differentiation or proliferation following activation of a signalling cascade regulating the transcription of both immediate and delayed early response genes. Once cells have entered the S-phase, they become refractory to a series of growth-factor-induced stimuli. The next cell cycle events are controlled by an intrinsic programme regulating the progression through the G_2_- and M-phases.

Similarly to many other malignancies, CMM cells progress through deregulation of control mechanisms ruling both cell proliferation and escape from programmed apoptosis [79]. Each cell cycle step is normally controlled by the expression of a precise set of regulatory molecules. Heteroprotein dimers combining a protein kinase and a regulatory cyclin form the basic clockwork of the cell cycle progression. A series of cyclins bind and activate cyclin-dependent kinases. The diverse cyclins C, D1 (CCND1 gene product), D2, D3, and E as well as CDK2, p16^INK4a^, p21^CIP1^, and p27^KIP1^ drive the cell cycle of proliferation in its progression during the G_1_- to the S-phase. Cyclin A normally regulates the passage from the S- to G_2_-phase, and cyclin B from the G_2_-phase to mitosis [80].

Melanocytic nevi rarely express cyclin A, B, D1, and D3. By contrast, these cyclins are commonly present in CMM [80]. In some CMM, a negative correlation was pointed out between cyclin A expression and the disease-free survival [81]. The cyclin B and D1 prognostic relevance remains unsettled in CMM. Of note, the GG-CCND1 mutation (A8706-CCND1 polymorphism) in peripheral blood cells represents a genetic predisposition for CMM. Increased cyclin D3 was reported to be associated with early relapse and decreased survival in thin CMM, but not in thicker CMM [82]. Cyclin E expression appeared to be negatively related with survival of CMM patients [10, 83].

 Cyclin-dependent kinase inhibitors downregulate progression through the cell cycle of proliferation [10, 80]. For instance, p16 normally inactivates cyclin D/CDK4 complexes in most melanocytic nevi [84–86]. By contrast, p16 expression is lost in most of the invasive, recurrent, and metastatic CMM [84–87]. This feature appears to be associated with decreased survival, although it does not seem to represent an independent prognostic parameter [80].

The p21 protein is rarely present in melanocytic nevi. By contrast, it shows increased immunoreactivity in CMM [88]. It inhibits cyclin/CDK complexes, binds to PCNA and thus, it inhibits DNA polymerase *δο*. Any relationship between increased p21 immunoreactivity and CMM outcome remains unsettled.

The p27 protein inhibits both cyclin D/CDK4 and cyclin E/CDK2 complexes. Thus, it blocks the cell cycle progression from the G_1_- to S-phase [89, 90]. A clear p27 expression distinction is not established between melanocytic nevi and CMM. Thick CMM with a p27 index lower than 5% might be at increased risk for early relapse. However, the magnitude in p27 expression has no effect on the overall survival [88].

 The p53 gene is commonly mutated in cancers. The normal wild-type p53 protein is a 53-kDa tumor suppressor protein blocking the cell cycle at both the G_1_- and G_2_-phases, allowing repair of the DNA damage [91]. In addition, p53 induces the expression of p21 contributing to inhibit DNA synthesis [88]. Mutations of the p53 gene produce abnormal p53 proteins unable to inhibit the cell cycle. As the regular p53 protein is short lived, it is not detected using immunohistochemistry. By contrast, the long-lived mutated p53 protein is conveniently disclosed using immunohistochemistry. Accordingly, p53 protein is not revealed in most melanocytic nevi, but is present in its mutated form in 25–60% of CMM [14, 88, 91–93]. Overexpression of the p53 protein was reported in CMM originating from a precursor p53-negative melanocytic nevus [92]. Some benign melanocytomas show nearly 10% cell positivity for the mutated p53 protein [94]. A correlation was disclosed between p53 expression and thicker CMM [95]. However, no correlation was found between p53 immunoreactivity and likelihood of metastasis, recurrence, and global CMM survival.

HDM2 is a 90-kDa zinc finger protein that binds to the transcription activation domain of the p53 gene [96, 97]. Increased HDM2 immunostaining might represent an independent prognostic factor paradoxically associated with decreased recurrence rates and increased survival in CMM [97].

## 9. Mitogen-Activated Protein Kinase Pathway in CMM

Gene expression profiles characterize cells from any given tissue. Alterations of these patterns potentially disrupt cell homeostasis leading to some diseases including cancer. Compared to regular melanocytes, a number of primary and secondary mutations were reported in advanced CMM. A primary clonal event in neoplastic progression possibly contributes to the malignancy. This process is possibly based on a genetic origin (gene mutation, deletion, amplification, or translocation). Such defect occurs independently, rather than as a secondary event following some other oncogenic changes. Alternatively, epigenetic alterations possibly result from heritable changes generally corresponding to transcriptional modulation following DNA methylation, and/or by chromatin alterations resulting from histone alteration. Clonal evolution of cancer initiates new clones exhibiting growth advantage over other cells, or an alternative selective advantage such as migration potential [98].

The gene products subject to primary clonal alterations in CMM correspond to activated or amplified genes, and conversely to other inactivated or deleted genes. Hundreds of secondary changes were described in CMM [99–103]. However, there are limitations to the interpretations of reported data. Indeed, genes were often tested only for mutations, rather than for deletions or amplifications. Hence, frequencies of aberration are probably underestimated [16]. In addition, some studies focused on cell lines, while others used uncultured native tissues. Both procedures commonly provided different results.

 A number of signalling pathways are critical to the survival and growth of CMM cells. The most important pathway is the mitogen-activated protein kinase (MAPK) cascade. It includes the MAPK kinase (MEK) which is just downstream BRAF, the extracellular signal-regulated protein kinase (ERK), and the p38 MAPK and the Jun NH2-terminal protein kinase (JNK) activation pathways [104]. MAPK activation is linked to the induction of the transcription factor AP-1 regulating the expression of a series of genes involved in the regulation of both cell growth and differentiation. The MAPK pathway is activated and altered in nearly all CMM. It is involved in the neoplastic proliferation, invasion, and survival [105, 106] through the RAS/RAF/MEK/ERK signal transduction pathway regulating cell growth.

MAPK signalling is initiated at the cell membrane, either by tyrosine kinases (RTKs) binding ligand or by integrin adhesion to the extracellular matrix. This latter event activates the RAS-GTPase at the cell membrane inner surface [107, 108]. GTP-bound RAS binds effector proteins, boosting cell proliferation, differentiation, and survival through activation of various signalling pathways [108]. RAF and phosphatidylinositol 3-kinase (PI3K) are well-characterized RAS effector proteins.

The RAF protein family is composed of serine/threonine kinases. It includes three proteins, ARAF, BRAF, and CRAF (corresponding to RAF-1) coded by unique genes [109–111]. Any RAF family member activates the MAPK pathway, although each isoform possesses a distinct expression profile with unique phosphorylation targets and signalling effects [110]. RAF is the primary link between RAS and the MAPK pathway. RAF has long been identified as a protooncogene [112]. It activates the cascade of proliferative or survival signals through phosphorylation of a number of cytoplasmic targets [14, 113].

 Over 50 BRAF mutations have been identified inside the kinase domain. The most common is the replacement of glutamic acid by valine in the position 600, the so-called V600E BRAF mutation [114–117]. This single aminoacid substitution accounts for 80–90% of the BRAF gene mutations [109, 112, 118–121]. In addition, RAS is mutated in approximately 15% of cancers including CMM [109]. The V600E mutant expresses a 10-fold increased kinase activity compared to the wild-type BRAF [109].

BRAF is mutated in about 7–30% of human cancers, but this mutation is present in almost 60–80% of CMM [107–111, 113, 119, 122]. BRAF mutations were found in a number of melanocytic nevi as well [109, 120, 121]. The presence of BRAF mutations in melanocytic nevi suggests that a single BRAF mutation in the MAPK pathway as found in CMM is probably inadequate to promote malignant transformation [109, 119, 120, 123, 124]. Rather a single BRAF mutation appears to represent a senescence factor in melanocytes and melanocytic nevi [124]. Another oncogenic hit is therefore necessary including the interaction of BRAF with PTEN [125], p16 [124], p53 [123], AKT [126], and UV radiation [127]. The NRAS and BRAF mutations present in CMM exhibit some characteristic UV radiation-induced changes. The target of UV injury leading to such mutations remains unclear [113, 128].

Clearly, histopathologic assessments point to the fact that the majority of CMM occur outside a precursor melanocytic lesion [128]. In general, CMM originating from a melanocytic nevus exhibit both a BRAF mutation or are both negative for the mutation [129, 130].

 In primary CMM, the genomewide alterations in DNA copy number, as well as BRAF and NRAS mutations [131], suggest the role of BRAF kinase both in CMM development and CMM heterogeneity [14]. Increased BRAF mutations are found in both nodular and superficial spreading CMM, contrasting with acral lentiginous CMM, lentigo maligna, and mucosal CMM [132–134]. CMM with the highest prevalence of BRAF mutations were those associated with intermittent sun exposure [132]. This relationship reveals a stringent relationship between sporadic UV exposure, BRAF/NRAS mutations, and additional genetic events in CMM [80, 122]. CMM without either mutation often exhibit increased copies of CDK4 or CCND1. Furthermore, no CMM with CDK4 amplification exhibited concomitant NRAS or BRAF mutations, or CCND1 amplification. Such finding suggests overlapping functions of the MAPK pathway and the CCND1/CDK4 pathways with independent oncogenic functions. The overall incidence of BRAF and NRAS mutations was significantly lower in CMM developed on chronic sun-damaged (CSD) skin as well as on sun-shielded sites, with BRAF and NRAS mutations being mutually exclusive. The CMM thickness had apparently no influence on frequency of mutation in BRAF or NRAS, or amplification (CCND1 or CDK4). In CMM developed on CSD, BRAF mutations were rare and CCND1 copy gain predominated [135]. Conversely, in CMM developed outside CSD, mutant BRAF and chromosome 10 (site of PTEN) loss were both common.

 An important link between germline mutations of the melanocortin-1 receptor (MC1R) and BRAF mutations is possible [136]. Although MC1R variants were identified as risk factors for CMM [137], the precise link to sun exposure and genetic events in primary CMM remains unclear. MC1R variant alleles were found to be associated with CMM risk, specifically in CMM developed outside CSD. Such risk was associated with neoplasms harbouring BRAF mutations suggesting that germline events largely influenced genetic events leading to tumorigenesis in response to environmental UV exposures.

 The enhancement of mitogenic activity in skin cancers is possibly reflected by the difference of intrinsic mitogenic signalling pathways [138]. A signalling pathway involves activation of the MAPK family, whose molecular components play a complex role in the determination of cell growth. It appears that p38 MAPK is activated by UV irradiation, cytokines, hormones, and some stresses such as osmotic shock and heat shock. Its prognostic value is possible in some malignancies [139].

## 10. Apoptosis in CMM

Programmed cell death or apoptosis is different in its self-destruction from necrotic cell death. It represents one major mechanism involved in reducing the expansile growth of melanocytic neoplasms. As a functional counterpart of mitosis, apoptosis plays a crucial role and is normally firmly regulated. Apoptosis is altered in melanocytic neoplasms when the components and regulators of the cellular apoptotic machinery are mutated or present in inappropriate amounts. In CMM, the molecular apoptosis machinery includes positive (proapoptotic) and negative (antiapoptotic) regulators [140–143]. The former includes p53, Bid, Noxa, PUMA, Bax, TNF*α*, TRAIL, Fas/FasL, PITSLRE, interferons (IFN), and c-KIT/SCF. The antiapoptotic regulators include Bcl-2, Bcl-X_L_, Mcl-1, NF-_K_B, survivin, livin, AKT, and ML-LAP [91, 94, 144]. Alternatively, some molecules such as TRAF-2, c-Myc, endothelin, and integrins either exhibit pro- or antiapoptotic effects. In addition, the PI3K pathway is activated following the biding of a ligand to a receptor tyrosine kinase (RTK). It interacts with multiple cellular mechanisms of apoptosis, survival, proliferation, mobility, differentiation, and growth. The phosphatase and tensin homolog (PTEN) is a tumor suppressor gene. Its protein product inhibits CMM growth and increases its susceptibility to apoptosis [145]. The deletion or silencing of PTEN increases the level of AKT3 phosphorylation in melanocytes and early-stage CMM cells.

 A small CMM subset (2 to 5%) presents amplification or mutation in c-Kit. It is more frequent in sun-shielded areas, such as in the mucous and acral CMM types, although it can be found on CSD areas [146, 147].

Bcl-2 is a protooncogene that is not involved in the mechanisms of cell proliferation, but instead influences tissue homeostasis regulated by apoptosis. The gene encodes for a protein that preserves cells from apoptosis, allowing them to survive in G_0_-phase even in the absence of essential growth factors. The Bcl-2 protein is conveniently detected using immunohistochemistry. It is expressed in most CMM and melanocytic nevi [148] suggesting that its expression is a common finding in cutaneous melanocytic tumors regardless of their biologic behavior. Expression of the Bcl-2 protein in the vast majority of CMM rules out any prognostic significance of Bcl-2 in CMM.

Some of the apoptosis-related molecules are of potential therapeutic use, including (a) p53, which influences resistance to chemotherapy, (b) Mcl-1 and Bcl-X_L_, which override apoptosis, (c) TRAIL, which has selective fatal effects on neoplastic cells, (d) downregulated NF-_K_B, which sensitizes cells to TRAIL and TNF, (e) PITSLRE kinases, whose alteration appears to result in Fas resistance, (f) IFN sensitizing cells to other factors, and (g) survivin that inhibits apoptosis.

 Impaired regulation of apoptosis is associated with the development of various cancers. Fas binding to its ligand, Fas ligand (Fas-L), is expressed by CMM cells and plays a role in tumoral escape from immune surveillance [141, 149]. Apoptotic activity appeared minimal in CMM and moderate in Spitz melanocytomas. By contrast, melanocytic nevi demonstrated more intense apoptosis in the deep portion of the tumor. Fas was found to be expressed by all Spitz melanocytomas, most melanocytic nevi, and approximately half of the CMM. Fas expression was significantly more pronounced in Spitz melanocytomas as compared with the two other neoplasms. Fas-L was shown to be more expressed and more frequent in CMM cells as compared to nevus cells.

## 11. Conclusion

When CMM frequency emerged a few decades ago, dermatologists, dermatopathologists, and oncologists clearly underestimated the impact and relevance of CMM cell proliferation including CMM stem cells. These limitations were obstacles to tackling CMM beyond the stage of the primary neoplasm often curable by surgery alone. With the improvement of dermatopathology and molecular biology beyond routine histopathology, skilled researchers were able to better assess the neoplastic CMM progression. They tackled many facets of CMM with both increased knowledge and the benefit of hindsight. There is evidence that future CMM treatments will target some specific molecular steps of the neoplastic cell cycle of proliferation and the apoptosis pathways.

 The links between the clinical evolution, cell proliferation/apoptosis, and key molecular aberrations are progressively elucidated in CMM. These advances represent key factors supporting the identification and distinction of different CMM types, including the growth-stunted, the slow-growing accretive growth and the fast-growing expansile proliferative neoplasms.
